# Molecular mechanisms underlying phenotypic degeneration in *Cordyceps militaris*: insights from transcriptome reanalysis and osmotic stress studies

**DOI:** 10.1038/s41598-024-51946-3

**Published:** 2024-01-26

**Authors:** Chinh Q. Hoang, Giang H. T. Duong, Mai H. Tran, Tao X. Vu, Tram B. Tran, Hang T. N. Pham

**Affiliations:** 1 Center of Experimental Biology, National Center for Technical Progress, C6 Thanh Xuan Bac, Thanh Xuan, Hanoi, Vietnam; 2 Center for Biomedical Informatics, Vingroup Big Data Institute, and GeneStory JSC, 458 Minh Khai, Hai Ba Trung, Hanoi, Vietnam; 3 GeneStory JSC, 458 Minh Khai, Hai Ba Trung, Hanoi, Vietnam; 4 Department of Pharmacology and Biochemistry, National Institute of Medicinal Materials, 3B Quang Trung, Hoan Kiem District, Hanoi, 100000 Vietnam; 5 University of Medicine and Pharmacy, Vietnam National University, 144 Xuan Thuy, Cau Giay District, Hanoi, 100000 Vietnam; 6 Present Address: Department of Molecular Biotechnology, Institute of New Technology, Academy of Military Science and Technology, 17 Hoang Sam, Cau Giay, Hanoi, Vietnam

**Keywords:** Developmental biology, Microbiology, Molecular biology

## Abstract

Phenotypic degeneration in *Cordyceps militaris* poses a significant concern for producers, yet the mechanisms underlying this phenomenon remain elusive. To address this concern, we isolated two strains that differ in their abilities to form fruiting bodies. Our observations revealed that the degenerated strain lost the capacity to develop fruiting bodies, exhibited limited radial expansion, increased spore density, and elevated intracellular glycerol levels. Transcriptome reanalysis uncovered dysregulation of genes involved in the MAPK signaling pathway in the degenerate strain. Our RT-qPCR results demonstrated reduced expression of sexual development genes, along with upregulation of genes involved in asexual sporulation, glycerol synthesis, and MAPK regulation, when compared to the wild-type strain. Additionally, we discovered that osmotic stress reduced radial growth but increased conidia sporulation and glycerol accumulation in all strains. Furthermore, hyperosmotic stress inhibited fruiting body formation in all neutralized strains. These findings indicate dysregulation of the MAPK signaling pathway, the possibility of the activation of the high-osmolarity glycerol and spore formation modules, as well as the downregulation of the pheromone response and filamentous growth cascades in the degenerate strain. Overall, our study sheds light on the mechanisms underlying *Cordyceps militaris* degeneration and identifies potential targets for improving cultivation practices.

## Introduction

Phenotypic switching, commonly referred to as phenotypic degeneration, is a well-documented phenomenon in fungi. When cultivated under artificial conditions or subjected to repeated subculturing on artificial media, fungi can undergo morphological alterations, such as growth retardation, changes in pigmentation, decreased virulence, and reduced production of secondary metabolites. These variations from batch to batch have significant implications for the quality and quantity of fungus-based products, making degenerate cultures a major concern for manufacturers^1^. While genetic and epigenetic factors can contribute to degeneration, with certain strains being more susceptible than others and specific gene mutations associated with phenotypic instability^2–4^, the cultivation media also play a role in this phenomenon. For instance, nutrient-rich cultures can accelerate the appearance of degenerative traits^5^.

Fungi, like all living organisms, possess the ability to sense and respond to changes in their environment, and the mitogen activated protein kinases (MAPK) pathway is one of the key signaling pathways they employ to adapt to these environmental cues. The MAPK pathway is a conserved signaling pathway found in all eukaryotic cells. It consists of a series of protein kinases that become activated in response to environmental signals. These kinases subsequently phosphorylate downstream targets, including transcription factors, which ultimately modulate gene expression to enable adaptation to new conditions^6^. Fungi employ the MAPK pathway for various processes, including cell cycle control, reproduction, morphogenesis, stress responses, cell wall assembly and integrity, virulence, and immunity^7^. In the yeast *S. cerevisiae*, there are five branches of the MAPK pathway that mediate responses to different intrinsic and extrinsic clues, including response to pheromones during mating (pheromone response—PR), filamentous growth under nitrogen starvation conditions (filamentous growth—FG), response to osmotic stress and other stress conditions (high-osmolarity glycerol—HOG), response to cell wall stress (cell wall integrity—CWI), and spore formation (SF)^8^. Mutations in two components of the MAPK pathway, *PaASK1* and *CpBck1*, have been linked to phenotypic instability in *Podospora anserine*^9^ and to sectorization in *Cryphonectria parasitica*^10^, but the precise role of the MAPK pathway in phenotypic degeneration remains poorly understood.

The MAPK pathways initiate signal transduction through G-Protein-Coupled Receptors, which detect intrinsic and extrinsic signals and transmit them to MAPK mediators like CDC42 and STE20 for the PR, FG, and HOG modules, RHO1 for the CWI cascade, and unidentified factor(s) for the SF branch. These MAPK regulators then activate MAPK kinase kinases such as STE11, which phosphorylate MAPK kinases to activate MAPKs like HOG1 and FUS3. The MAPKs then translocate to the nucleus and modulate the activity of transcription factors such as STE12 in the PR and FG modules, or HOT1 in the HOG cascade, initiating transcriptional programs that facilitate adaptation to new environments^8^.

The PR, FG, and HOG modules share certain components but elicit distinct responses to different extracellular stimuli, indicating the existence of cross-pathway regulatory mechanisms. For instance, both the FG and PR branches utilize the transcription factor STE12 to initiate transcriptional responses, but the STE12 and TEC1 heterodimer specifically induces genes involved in filamentous growth^8^. When the fungus responds to pheromone peptides, the MAPK FUS3 is activated and translocated to the nucleus, leading to the activation of STE12 and the inhibition of TEC1. This prevents the expression of genes related to filamentous growth, thereby fine-tuning the mating pathway signal^11,12^. Similarly, hyperosmotic stress triggers the activation of the HOG cascade, which inhibits TEC1 activity and fine-tunes the stress response pathway^13^. The presence of Hog1 and Pbs2 genes, which are components of the HOG module, is crucial for preventing the activation of the PR cascade by osmotic stress^14^. Moreover, the activated HOG branch partially inhibits the mating response^15^, and delays or attenuates the PR cascade^16^.

*Cordyceps militaris (C.* *militaris)* is an ascomycete fungus renowned for its medicinal properties, including immune modulation, anti-inflammation, anticancer effects, and treatment of conditions such as diabetes, stroke, and cardiovascular diseases. It has been utilized in traditional medicine in Asia for centuries and has gained popularity in Europe and America in recent decades^17,18^. To meet the demands of the market, *C. militaris* is cultivated under artificial conditions. However, strain degeneration during subculturing and preservation poses a significant challenge for manufacturers. Degenerated cultures display reduced growth rates, impaired fruiting body formation, altered pigmentation, and/or diminished production of secondary metabolites^19^.

Degenerate strains of *C. militaris* are associated with genetic and epigenetic changes, including mutations in the 18S and mating-type regions of the fungus^20^. These degenerate strains also exhibit significantly higher levels of DNA methylation, with differentially methylated genes enriched in pathways related to pyruvate metabolism, glycerophospholipid metabolism, DNA replication, and N-glycan biosynthesis^21^. Changes in gene expression are also linked to degeneration, with over 2000 differentially expressed genes identified in degenerate strains, including genes involved in toxin biosynthesis, energy metabolism, DNA methylation, and chromosome remodeling^22^. These findings suggest that environmental alterations may induce genetic and epigenetic changes that contribute to phenotypic degeneration in *C. militaris* strains. However, the specific genes and pathways responsible for the degenerate phenotypes remain unknown.

In this study, we isolated two strains exhibiting contrasting abilities to form fruiting bodies and subsequently analyzed their phenotypes to identify easily quantifiable characteristics. Additionally, we conducted a transcriptome reanalysis of previously published gene expression data to discern the biological pathways associated with degeneration. Using Quantitative reverse transcription polymerase chain reaction (RT-qPCR), we examined dysregulated genes linked to the phenotypes observed in our degenerate *C. militaris* strain. Moreover, we performed manipulations on a candidate pathway to confirm its involvement in phenotypic degeneration. The primary objective of our study is to enhance our understanding of the molecular mechanisms underlying phenotypic degeneration in fungi and to identify potential targets for preventing or mitigating its effects.

## Results

### Identification of phenotypic degeneration indicators in *C. militaris*

The degenerate strain of *C. militaris* is characterized by the loss or reduction of fruiting body formation, which becomes apparent after approximately two months. Consequently, we aimed to identify rapid and reliable indicators of degeneration that are easily measurable and quantifiable. In our experiments, we found that radical expansions and spore density can be conveniently assessed after several days of culture on potatoes dextrose agar (PDA) plates. Therefore, we propose to use these two characteristics as indicators of phenotypic degeneration in future studies of *C. militaris*.

**Figure 1:**
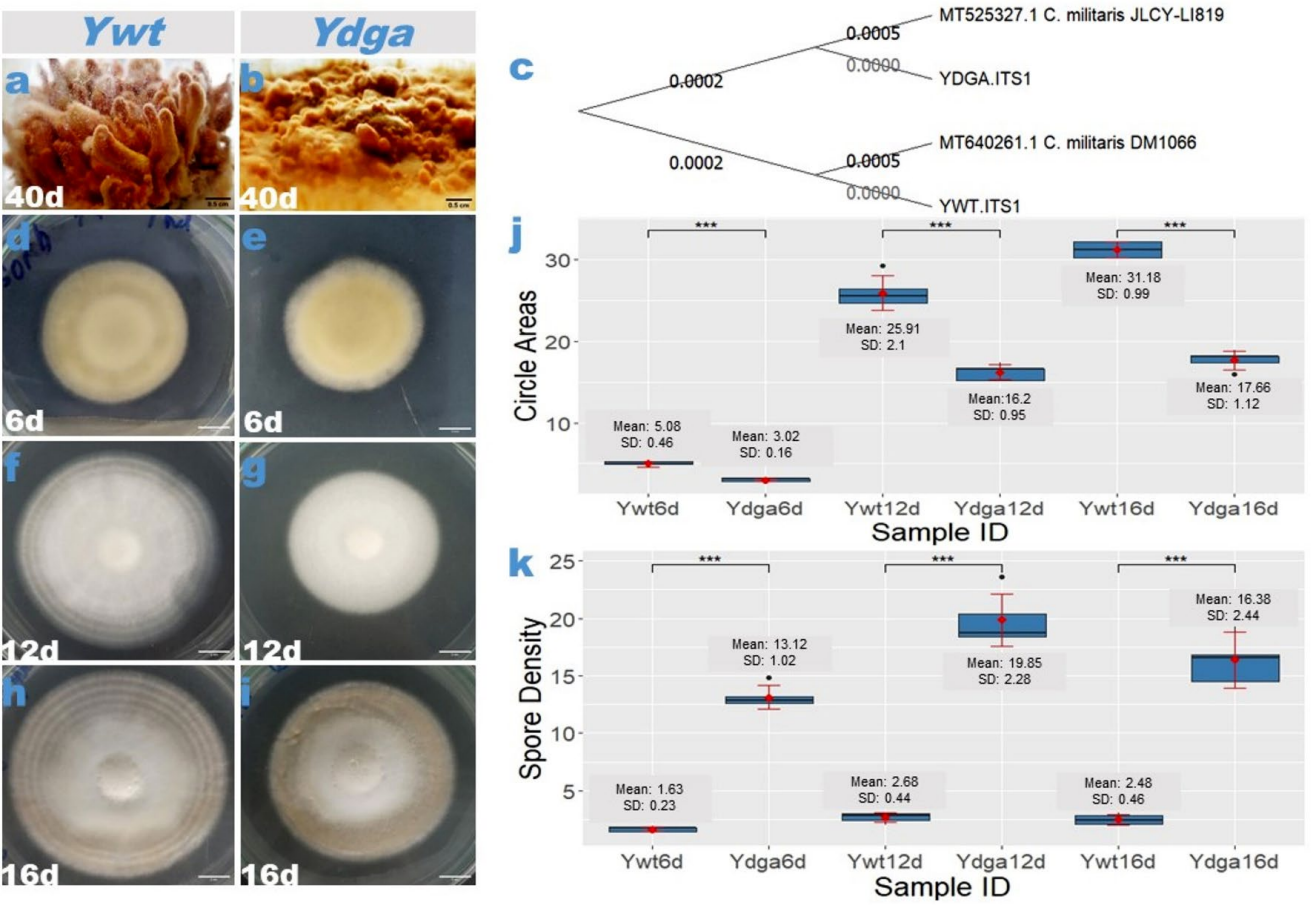
Characteristics of *C. militaris* phenotypic degeneration. (**a** vs.** b**) Comparison of fruiting body development between the neutralized strain (*Ywt*) and the degenerate strain (*Ydga*), illustrating that the former successfully develops fruiting bodies (**a**), the latter fails to do so (**b**). (**c**) Phylogenetic tree depicting the relationship among different *C. militaris* strains. Representative images of *C. militaris* isolates (*Ywt* vs. *Ydga*) cultured on PDA plates for 6 (d vs. e), 12 (**f** vs. **g**), and 16 (**h** vs. **i**) days, providing visual evidence of the phenotypic differences. Statistical analysis revealed considerable variations in circle areas (**j**) and spore densities (**k**) between the two *C. militaris* variants (*Ywt* vs*. Ydga*). The data are presented as ranges, means, and standard deviations, with a sample size of n = 5. *** represents a significant difference (actual *p* values are presented in supplementary Table S2–S3). SD refers to standard deviation. Sample ID indicates the strain name and the number of culture days. Significance was determined using a one-tailed t-test for two independent means, with an alpha level of 0.05.

We isolated two variations of the *C. militaris* fungus from the same batch of cultivation and conidial origin. One of these isolates, named *Ywt* (Fig. 1a), is capable of forming fruiting bodies, while the other, named *Ydga* (Fig. 1b), cannot. To confirm the genetic identity and relationship between the two variants, we conducted a BLAST search and phylogenetic analysis using nucleotide sequences of the internal transcribed spacer (ITS) region of nuclear ribosomal DNA. Our analysis revealed that the ITS sequences of *Ywt* and *Ydga* (Supplementary Table S1) were identical to those of most, if not all, *C. militaris* specimens deposited in the National Library of Medicine, National Center for Biotechnology Information (NCBI). Moreover, the neighbor joining tree analysis demonstrated that *Ywt* and *Ydga* were closely related to other reported *C. militaris* strains (Supplementary Fig. S1).

To gain a better understanding of the relationship between our *C. militaris* strains and other known strains, we compared them to the *DM1066* and *JLCY-LI819* strains through phylogenetic analysis. The analysis showed that *Ydga* and *Ywt* were closely related to a common ancestor of the four strains, with a small interior branch length, while *DM1066* and *JLCY-LI819* were more distantly related (Fig. 1c). This suggests that *Ydga* and *Ywt* likely share an identical genetic background.

To evaluate whether phenotypic degeneration affects hyphal development and conidiogenesis, we compared the radical expansion and spore density of the *Ywt* strain with those of the *Ydga* strain at three different time points: 6, 12, and 16 days of culturing. At the 6-day culture mark, the appearance of *Ydga* colonies was similar to that of *Ywt*, except for slower hyphal growth (Fig. 1d vs. e). However, at the 12- and 16-day cultures, the hyphae of *Ydga* appeared irregular and fluffy, whereas those of *Ywt* were smooth and ring-shaped at the colony edge (Fig. 1d–g). Statistical analysis revealed that *Ydga* exhibited approximately two times slower radical expansion compared to *Ywt* at all examined time points (Fig. 1J, Supplementary Table S2), indicating a lower growth rate and a defect in hyphal development. Additionally, statistical analysis showed that *Ydga* had almost ten times higher spore density than *Ywt* at all examined time points, suggesting increased conidial formation (Fig. 1K, Supplementary Table S3).

Overall, these findings suggest that retardation of hyphal development and an increase in sporulation may persist as characteristic features of phenotypic degeneration, which can be easily observed after several days of culturing on PDA plates.

### Transcriptome analysis reveals dysregulated MAPK signaling pathway in the culture degeneration of *C. militaris*

**Figure 2:**
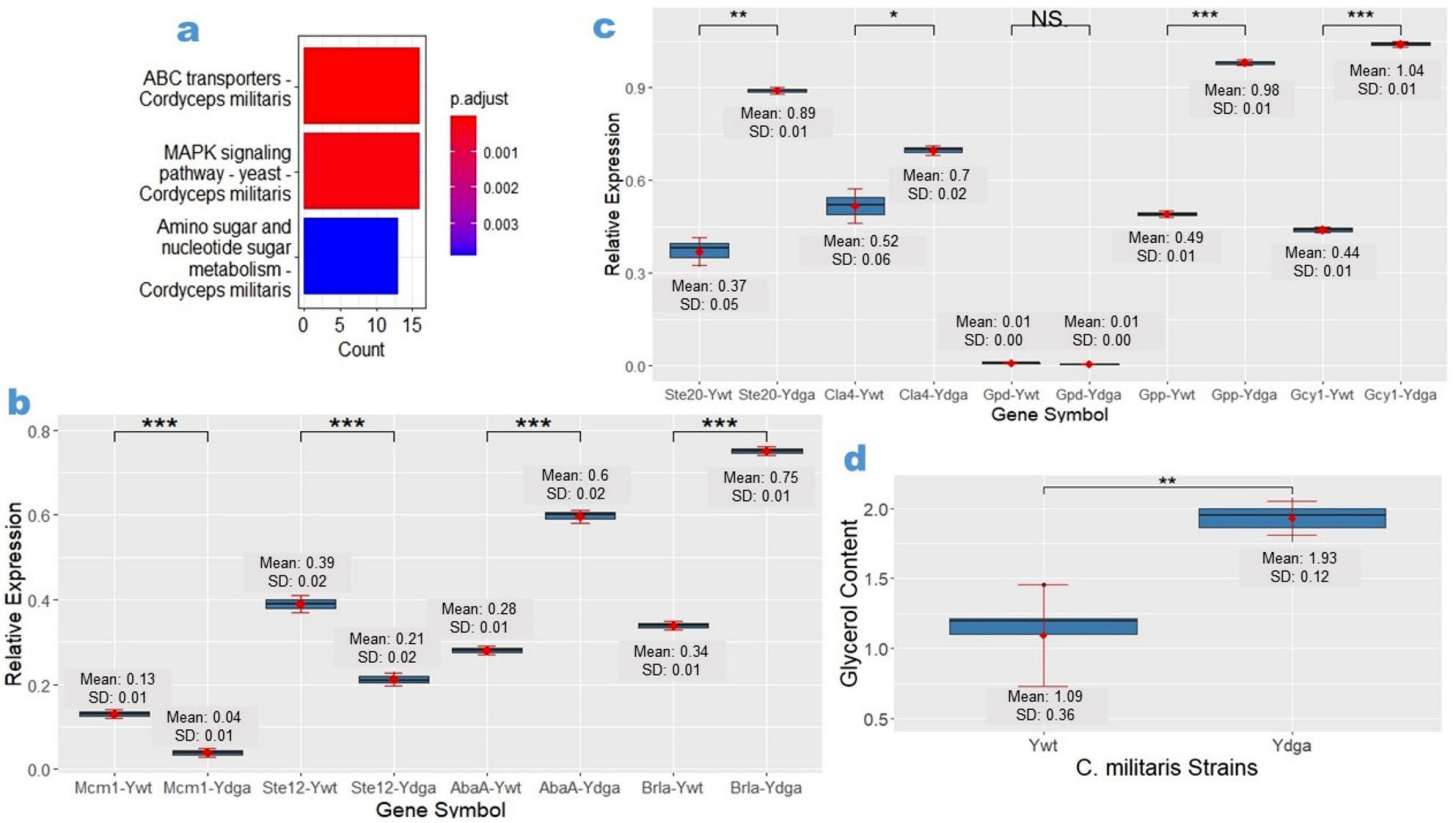
Dysregulation of the MAPK signaling pathway in *C. militaris* phenotypic degeneration. (**a**) Biological pathways considerably enriched in downregulated genes. (**b**,**c**) RT-qPCR expression analysis of genes involved in the MAPK pathway and associated with phenotypic degeneration, including genes related to sexual development, sporulation, and glycerol synthesis in the *Ywt* and *Ydga* strains. (**d**) Comparison of intracellular glycerol contents between *Ywt* and *Ydga*. The data are presented as ranges, means, and standard deviations, with a sample size of n = 5 for intracellular glycerol contents and n = 3 biological replicates for RT-qPCR. *, **, *** represents a significant difference (actual *p* values are presented in supplementary Table S5–S6). SD refers to standard deviation. Gene symbols indicates the tested genes in the corresponding strains. Significance was determined using a one-tailed t-test for two independent means, with an alpha level of 0.05.

In order to investigate the underlying molecular mechanisms of culture degeneration in *C. militaris*, we conducted a transcriptome analysis by comparing the gene expression profiles of a degenerate strain with those of a wild-type strain. Our analysis identified 880 downregulated genes and 1034 upregulated genes (False Discovery Rate < 0.05) in the degenerate strain. A detailed list of differentially expressed genes is presented in the file 'DEgenes.SR-Hoang et al.', which is available for reference. Among the downregulated genes, we observed considerable enrichment in gene ontology terms associated with ABC transporters, MAPK signaling pathway, and amino sugar and nucleotide sugar metabolism (Fig. 2A, Supplementary Table S4), while no biological pathway was found to be significantly enriched among the upregulated genes.

Given the importance of the MAPK signaling pathway in regulating cellular processes such as sexual development and stress responses, which are known to be affected by degeneration, we focused our attention on this pathway. To validate our findings, we performed RT-qPCR analysis to examine the expression levels of key genes involved in the MAPK pathway and associated with the phenotypes of our *C. militaris* degenerate strain. Specifically, we investigated genes related to sexual development (*Ste12, Mcm1*), asexual sporulation (*Brla, AbaA*), glycerol synthesis (*Gcy1, Gpd,* and *Gpp*), as well as MAPK regulators (*Ste20, Cla4*) in the *Ywt* and *Ydga* strains. Our analysis revealed that compared to the wild-type strain *Ywt*, the degenerate strain *Ydga* exhibited lower expression of sexual development genes (*Ste12* and *Mcm1*), but showed higher expression of asexual sporulation genes (*Brla and AbaA*) (Fig. 2B, Supplementary Table S5). Furthermore, the degenerate strain exhibited increased expression of MAPK mediators (*Ste20* and *Cla4*) and glycerol-synthesizing enzymes (*Gcy1* and *Gpp*), which are typically activated in response to hyperosmotic conditions, while the expression of the basal glycerol-synthesizing enzyme (*Gpd*) did not show a subtantial difference (Fig. 2C, Supplementary Table S5). These findings were consistent with the higher intracellular glycerol content observed in the degenerate strain compared to the neutralized strain (Fig. 2D, Supplementary Table S6). Collectively, our results suggest dysregulation of the MAPK signaling pathway in the degenerate strain, which likely contributes to the observed phenotypic changes, including reduced radial growth, loss of fruiting body formation, increased conidia sporulation, and elevated intracellular glycerol levels. In summary, our study provides insights into the potential involvement of the MAPK signaling pathway in the phenotypic degeneration of C. militaris.

### The HOG module may play a role in *C. militaris* phenotypic degeneration

The dysregulated transcription of the MAPK signaling pathway components and the increased intracellular glycerol content observed in the degenerate strain of *C. militaris* led us to investigate the potential role of stress in the phenotypic degeneration, particularly focusing on the HOG module. We exposed both the wild-type strain (*Ywt*) and the degenerate strain (*Ydga*) of *C. militaris* to various MAPK activators and assessed their effects on radial growth, conidia sporulation, and glycerol accumulation.

**Figure 3:**
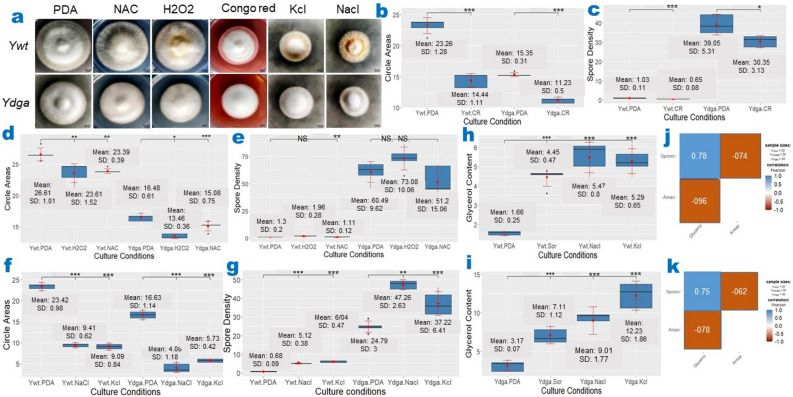
Effects of MAPK Activators on Radial Expansion, Sporulation, and Intracellular Glycerol in *C. militaris*. (**a**) Representative images of *C. militaris Ywt* and *Ydga* cultured in PDA with or without various MAPK activators. Congo red, a cell wall integrity (CWI) activator, considerably inhibited both radial expansion (**b**) and sporulation (**c**) in both strains of *C. militaris*. (**d**) H_2_O_2_, an oxidative stress inducer, and NAC, an antioxidant agent, substantially suppressed radial expansion in both strains. (**e**) H_2_O_2_ considerably increased spore density in *Ywt* strain but not in *Ydga* strain, while NAC had no substantial effect on sporulation in either strain. KCl and NaCl, two osmotic stressors, considerably inhibited radial expansion (**f**) but promoted sporulation (**g**) in both strains. Osmotic stressors substantially increased intracellular glycerol concentrations in *Ywt* (**h**) and *Ydga* (**i**). Glycerol concentrations exhibited a positive correlation with spore density but a negative correlation with circle areas in *Ywt* (**J**) and *Ydga* (**K**). CR = PDA + 200 μg/ml Congo Red. H_2_O_2_ = PDA + 0.04% H_2_O_2_; NAC = PDA + 200 mM* N*-AcetylCysteine. NaCl = PDA + 0.4 M NaCl; KCl = PDA + 0.4 M KCl; Sor = PDA + 1 M Sorbitol. The data are presented as ranges, means, and standard deviations, with a sample size of n = 5. *, **, *** represents a significant difference (actual *p* values are presented in supplementary Table S9–S11). SD refers to standard deviation. Culture Conditions indicates the strain name and the corresponding media. Significance was determined using a one-tailed t-test for two independent means, with an alpha level of 0.05.

Our results showed that Congo red, a stressor that affects cell wall integrity (CWI), had similar effects on both strains, leading to reduced radial growth and conidia sporulation (Fig. 3a–c, Supplementary Table S7). This suggests that the CWI branch is not strongly involved in the phenotypic degeneration of *C. militaris*. However, we observed differential effects of oxidative and osmotic stressors on the two strains. Hydrogen peroxide (H_2_O_2_), an oxidative stress inducer, and* N*-AcetylCysteine (NAC), an antioxidant agent, substantially suppressed radial expansion in both strains (Fig. 3a–d, Supplementary Table S8). Interestingly, H_2_O_2_ significantly increased spore density in the wild-type strain (*Ywt*) but not in the degenerate strain (*Ydga*), while NAC did not have a substantial effect on sporulation in either strain (Fig. 3e, Supplementary Table S8).

The most notable effects on both strains were observed when they were exposed to osmotic stressors such as KCl, NaCl, or sorbitol. These stressors considerably reduced radial growth but increased conidia sporulation and intracellular glycerol accumulation in both strains (Fig. 3a,f–i, Supplementary Table S9–S11). These findings suggest that osmotic stress may play a significant role in the phenotypic degeneration of *C. militaris*. Additionally, we observed positive correlations between intracellular glycerol concentrations and spore density, as well as negative correlations with radial expansion in *Ywt* (Fig. 3j) and *Ydga* (Fig. 3k). These observations indicate the presence of cross-regulatory mechanisms among the high-osmolarity glycerol (HOG), filamentous growth (FG), and spore formation (SF) modules. Collectively, our findings suggest that osmotic stress, potentially involving the HOG cascade, contributes to the phenotypic degeneration of *C. militaris*.

### Hyperosmotic stress induces phenotypic degeneration in neutralized *C. militaris* strains

**Figure 4:**
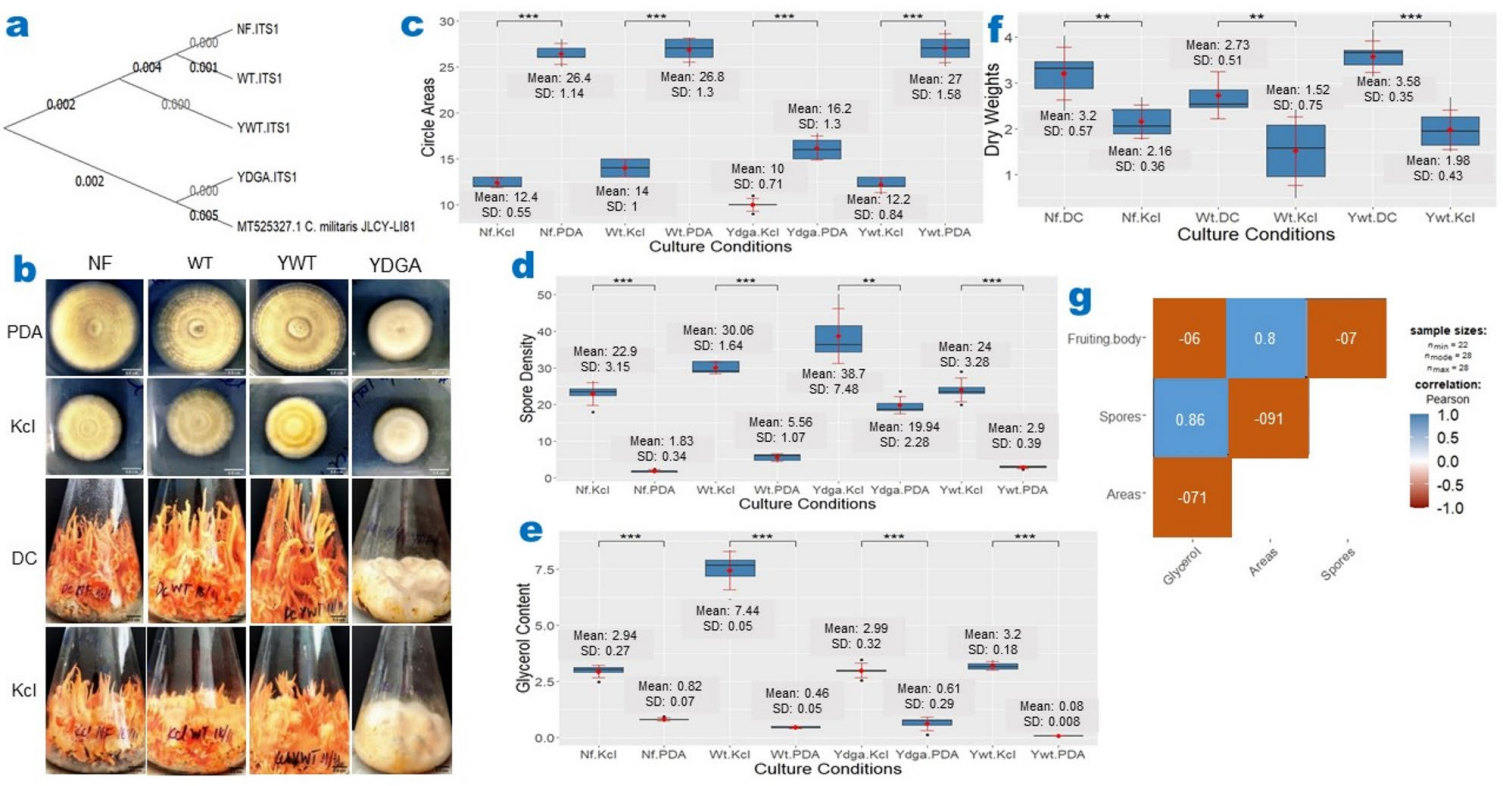
Induction of Phenotypic Degeneration in Neutralized *C. militaris* Strains by Hyperosmotic Conditions. (**a**) Phylogenetic tree depicting the relationship among *C. militaris* strains. (**b**) Representative images illustrating the repression of radial growth (upper panel) and fruiting body development (lower panel) under hyperosmotic culture conditions. Hyperosmotic conditions considerably suppressed radial expansion (**c**) and fruiting body development (**f**) while promoting increased spore densities (**d**) and intracellular glycerol concentrations (**e**) in all neutralized *C. militaris* strains. (**g**) Glycerol concentrations exhibited a positive correlation with spore density but a negative correlation with circle areas and fruiting body weights. PDA = Potato dextrose agar; DC = Fruiting Body Media (FBM); KCl = PDA or FBM + 0.4 M KCl. The data are presented as ranges, means, and standard deviations, with a sample size of n > / = 7. *, **, *** represents a significant difference (actual *p* values are presented in supplementary Table S12–S14). SD refers to standard deviation. NS refers to Nonsignificant. Culture Conditions indicates the strain name and the corresponding media. Significance was determined using a one-tailed t-test for two independent means, with an alpha level of 0.05.

To investigate whether the involvement of the HOG cascade in *C. militaris* phenotypic degeneration is a widespread phenomenon, we studied the effects of hyperosmotic stress on four *C. militaris* strains, including three neutralized strains (*Nf, Wt*, and *Ywt)* and one degenerate strain (*Ydga*). We confirmed the origin and phylogenetic relationship of the *Nf* and *Wt* strains with the *Ywt, Ydga* and *JLCY-LI819* strains by ITS DNA sequencing. The resulting phylogenetic tree indicated that these strains share a common ancestor and that the neutralized strains (*Ywt, Wt* and *Nf*) belong to the same clade (Fig. 4a, for ITS sequences see Supplementary Table S1). Our findings demonstrated that the addition of potassium chloride (Kcl) to PDA media decreased radial growth by approximately 50% in all examined strains (Fig. 4b—upper panel, Fig. 4c, Supplementary Table S12). Additionally, hyperosmotic stress increased spore production (Fig. 4d, Supplementary Table S12) and glycerol accumulation (Fig. 4e, Supplementary Table S13) in all strains. Interestingly, hyperosmotic stress inhibited fruiting body formation by approximately 40% in all neutralized strains, while the degenerated strain failed to produce fruiting bodies under any condition (Fig. 4A—lower panel, Fig. 4f, Supplementary Table S14).

Furthermore, we observed a positive correlation between glycerol concentration and spore density, and a negative correlation between glycerol concentration and radial growth area and fruiting body weight (Fig. 4g). Taken together, our results suggest that in *C. militaris* degenerate strains, the high-osmolarity glycerol (HOG) and spore formation (SF) modules may be activated, while the pheromone response (PR) and filamentous growth (FG) cascades may be deactivated, thereby contributing to the observed phenotypic degeneration.

## Discussion

*Cordyceps militaris*, a nutritionally and medicinally important ascomycete mushroom, faces significant challenges in long-term cultivation due to strain degeneration, which negatively impacts its commercial production. This study aimed to investigate the molecular mechanisms underlying the observed phenotypic degeneration in *C. militaris.*

The degenerate strain of *C. militaris* in this study exhibited several characteristics associated with degeneration. It lost the ability to produce fruiting bodies, displayed reduced radical expansion, and exhibited increased conidia density and intracellular glycerol content. To understand the underlying molecular changes, we analyzed transcriptome data and identified dysregulation of several molecular pathways, including the MAPK signaling pathway. The MAPK pathway is involved in the regulation of various cellular activities and has been associated with degeneration processes in other organisms. Through RT-qPCR analysis, we confirmed altered expression levels of key genes involved in the MAPK pathway, which correlated with the observed phenotypic characteristics of our degenerate *C. militaris* strain. To further investigate the specific branches of the MAPK pathway involved in degeneration, we subjected *C. militaris* strains to different stressors. Our results indicated that hyperosmotic stress induced phenotypic degeneration in neutralized *C. militaris* strains, and the activation of the HOG module may contribute to this degeneration.

Given that the loss or reduction of fruiting body formation is currently the sole indicator of *C. militaris* degeneration, we aimed to identify easily quantifiable morphological phenotypes that manifest within a shorter timeframe compared to fruiting body formation, which typically takes two months. For this purpose, we isolated two variations of *C. militaris* from the same cultivated batch: one capable of producing fruiting bodies and the other unable to do so on solid media. After culturing the strains on PDA plates, we compared their radical expansion and found that the degenerate strain exhibited approximately 50% reduced radical expansion compared to the wild-type strain. While a previous study by Wellhan et al.^23^ reported slightly slower radical growth rates in a degenerate strain, they did not investigate its ability to form fruiting bodies, making it unclear whether their “degenerate” strain truly exhibited degeneration. In our study, we also observed a significantly higher conidia density in the degenerate strain, which contradicted the findings of Meiyu et al.^24^ who reported a lower total number of conidia per dish in their degenerate strain by day 20 of culture. These discrepancies may stem from differences in the strains studied, the timing of sample collection, and variations in methodologies employed. Moreover, our findings indicate that the retardation of hyphal development and an increase in sporulation could be persistent features of phenotypic degeneration. This notion is supported by our observations that the degenerate strain (*Ydga*) displayed smaller circle areas and higher spore densities compared to the neutralized strain (*Ywt*) at three distinct time points during the developmental process.

To gain insights into the signaling pathways involved in the phenotypic degeneration of *C. militaris*, we conducted a reanalysis of transcriptome data generated by Yin et al.^22^. Our analysis identified approximately 2000 differentially expressed genes. Among the downregulated genes, we observed significant enrichment of pathways related to ABC transporters, MAPK signaling, and amino sugar and nucleotide sugar metabolism. In contrast, Yin et al.^22^ identified over 2000 differentially expressed genes, including those involved in toxin biosynthesis, energy metabolism, DNA methylation, and chromosome remodeling. This discrepancy may have arisen from differences in the bioinformatics pipelines and/or the datasets used for analysis.

Considering the significance of the MAPK signaling pathway in regulating diverse cellular processes, including sexual development and stress responses, and its association with phenotypic switching in other fungal species^7–9, 25^, we focused our attention on this pathway. To validate the expression levels of crucial genes involved in the MAPK pathway and their potential correlation with the observed phenotypes in our degenerate *C. militaris* strain, we employed RT-qPCR. Our RT-qPCR results showed consistent downregulation of sexual development genes (*Ste12* and *Mcm1*) and upregulation of conidiation genes (*BrlA* and *AbaA*) in the degenerate strain (*Ydga*) compared to the wild-type strain (*Ywt*). Additionally, the expression of MAPK-activator (*Ste20* and *Cla4*) and glycerol synthesis (*Gpp* and *Gcy1*) genes was significantly higher in *Ydga* compared to the wild-type strain (*Ywt*).

In our study, we discovered that the *C. militaris* orthologs of the yeast transcription factors STE12 and MCM1 exhibited downregulation. STE12, which is regulated by the PR module and involved in sexual development in response to pheromone signals, interacts with TEC1 to control filamentous growth under starvation conditions and collaborates with MCM1 to regulate the expression of pheromone-inducible genes necessary for proper sexual development^26–29^. However, the specific role of STE12 and STE12-like proteins in fruiting body formation varies between species^26^. MCM1 function is required for fruiting body development in the homothallic ascomycete *Sordaria macrospora*, as well as growth, conidiogenesis, cell wall integrity, and the cell cycle in the filamentous insect-pathogenic fungus *Beauveria bassiana*^30,31^. These findings suggest that the downregulation of *Ste12* and *Mcm1* orthologous genes in the *C. militaris* degenerate strain *Ydga* may contribute to growth and fruiting body retardation.

The central regulatory pathway for conidiogenesis, involving three sequentially controlled transcription factors (BRLA, ABAA, and WETA), is conserved in Ascomycete fungi^32^. In *Aspergillus*, BRLA is the essential activator of asexual sporulation, and its deletion results in failure to develop vesicle structures and instead produces elongated, bristle-like aerial stalks^33^. The deletion of *brlA* also abolishes the production of other conidiation-specific genes such as *AbaA, WetA, VosA* and *RodA*^34^. *AbaA* is required for the differentiation of phialides, and its loss of function results in the production of cylinder-like terminal cells with no conidia being formed. *WetA* functions in completing conidiogenesis, and its loss results in colorless conidia with defective spore walls^33^. This regulatory network, which is regulated by the Slt2-MAPK/RNS1, Fus3-MAPK and Hog1-MAPK cascades, also regulates asexual development in *Metarhizium*, and their loss of function impairs conidiogenesis^35^. These data suggest that upregulation of the *BrlA* and *AbaA* transcripts in the *C. militaris* degenerate strain *Ydga* may lead to an increase in its conidia density.

Glycerol synthesis and accumulation are known to play a significant role in responding to hyperosmotic and oxidative stresses^36^. In various organisms, such as *yeast* and *Aspergillus nidulans*, specific enzymes, such as GPD and GLD (orthologous to GCY1), respectively, are critical for adapting to hyperosmotic stress^37,38^. Similarly, in *Cryptococcus neoformans*, the enzyme GPP2 is essential for responding to different stresses^39^. In our study, we observed upregulated transcription levels of *Gcy1* and *Gpp* in the degenerate strain *Ydga*, while the difference in the expression of *Gpd* was not significant. These findings indicate that the increased glycerol content in the *Ydga* strain may be partially dependent on GLD and GPP enzymes rather than GPD. Furthermore, the *Ydga* strain showed increased expression of *Ste20* and *Cla4* transcripts, which are associated with glycerol biosynthesis enzymes in yeast. Collectively, these results suggest that the elevated glycerol levels observed in the *Ydga* strain might be attributed to enhanced expression of genes involved in glycerol synthesis and accumulation.

The degenerate strain *Ydga* exhibited higher glycerol concentrations and increased levels of glycerol biosynthesis enzymes, suggesting the activation of the HOG module in this strain. Supporting this notion, our experiments conducted under hyperosmotic conditions demonstrated that all three neutralized *C. militaris* strains experienced phenotypic degeneration, characterized by inhibited fruiting body and hyphal development, along with elevated conidia density and glycerol accumulation. Moreover, the growth of *C. militaris* radicals and the expression of the *Fus3* and *Hog1* genes were suppressed in a concentration-dependent manner under hyperosmotic conditions^40^. The findings from our study suggest that dysregulation of the MAPK signaling pathway and increased glycerol content may play a role in the phenotypic degeneration of *C. militaris*. Furthermore, the upregulation of conidiation-related genes and glycerol biosynthesis genes, typically activated in response to hyperosmotic conditions, along with the downregulation of sexual development genes, may contribute to the observed abnormalities in radical growth, sporulation, and fruiting body development. The positive correlation between intracellular glycerol and spore density, as well as the negative correlation with radial expansion and fruiting body development, provide additional support for these findings.

It is important to note some limitations of our study. First, we only focused on two morphological characteristics as indicators of phenotypic degeneration, and further investigations should consider additional indicators and criteria. Second, our findings may not be representative of all *C. militaris* strains, as we examined a single degenerate strain. Third, transcriptome analysis provides insights into gene expression changes but may not reflect protein expression or activity, necessitating further confirmation using alternative approaches. Fourth, while we focused on the MAPK signaling pathway, other molecular pathways may also be involved, warranting additional exploration. Finally, the stressors used in our study may not fully replicate natural environmental conditions, emphasizing the need for investigations under ecologically relevant conditions.

In summary, our study sheds light on the molecular mechanisms that contribute to phenotypic degeneration in *C. militaris*. The dysregulation of the MAPK signaling pathway and increased glycerol content observed in the degenerate strain indicate their potential involvement in this process. These findings carry implications for comprehending fungal physiology and morphology, improving the production of bioactive compounds, and developing early detection and monitoring methods for degenerative strains. However, further research is necessary to address the limitations and broaden our understanding of phenotypic degeneration in filamentous fungi.

## Methods

### Fungal strain and media

Strains of neutralized *C. militaris* (*Nf* and *Wt*) were gifted by Vu Duy Nhan and Le Hai Yen from the Laboratory of Macro Fungi Technology, Institute of Microbiology and Biotechnology, while strains of neutralized (*Ywt*) and degenerate (*Ydga*) *C. militaris* were isolated in our laboratory. The strains were cultured on potato dextrose agar (PDA) media containing 200 g/L potato, 20 g/L glucose, and 2 g/L agar, or potato dextrose broth (PDB) media containing 200 g/L potato and 20 g/L glucose. Fruiting body media (FBM) was prepared by mixing 35 g of brown rice with 70 mL of liquid media containing 200 g/L potato, 20 g/L glucose, and 100 g/L silkworm pupae.

### Phenotypic analysis

For the assessment of colony size and conidia density, the *C. militaris* strains were cultivated on PDA medium. A total of 10 µL containing 10^6^ conidia were placed at the center of a PDA Petri dish and incubated for the specified duration under natural daily dark–light cycles. The colony diameters were measured, and their corresponding areas were calculated using the formula {(diameter/2)^2^ × 3.14}, considering the colonies as circular in shape. The conidia were harvested in the sterile 0.01% Triton X-100 (MERK, cat# 9036-19-5) and filtered through milk filters (Lamtor Ltd, Bolton, UK) to remove hyphal fragments. The conidia were counted with a hemocytometer and their density was calculated using the formula [(conidia concentration × number of mL of the filtered suspension)/circles of areas of corresponding colony].

To measure fruiting body weight, the *C. militaris* conidia were cultured in PDB media at 130 rpm and 25 °C for 3 days. The inoculated media were then added to the FBM and incubated in the dark for 2 weeks, followed by 6 weeks in a 12/12 dark–light cycle with 90% humidity. The fruiting bodies were collected, dried at 80 °C, and weighed.

To assess hyperosmotic stress, the *C. militaris* strains were cultured on PDA, PDB, or FBM supplemented with 0.4 M KCl, 0.4 M NaCl, or 1 M sorbitol.

### Glycerol measurement

The *C. militaris* hyphae were collected from 6-day-old cultures on PDA plates, and their weight was recorded. Then, 1.2 mL of 50% EtOH was added to each tube, and the suspension was incubated in an ultrasonic bath with sonication at 70 °C for 15 min. One milliliter of the supernatant was then transferred to new tubes after centrifugation at 14,000 rpm (Eppendorf K-5418R). A total of 1.2 mL of a 10 mM sodium periodate solution (Merck, cat# 7790-28-5) was added to the suspension, and the mixture was shaken for 30 s. Then, 1.2 mL of a 0.2 M acetylacetone (Merck, cat# 123-54–6) solution was added to the former solution and kept in a water bath at 70 °C for 10 min. The sample absorbance was measured with a UV–Vis–NIR spectrophotometer (Cary 5000, version 3.00, Agilent, Scan Version 6.2.0.1588), and the glycerol concentrations were estimated using the formula Y = 0.0055*Ab-0.0012. [Y = glycerol concentration; Ab = absorbance at 413 nm. The formula was build based on a twofold serious dilution of glycerol (> / = 99% purification, Merck, cat # 56-81-5). The glycerol content (microgram glycerol/mg hyphal weight) was calculated by dividing the total glycerol content of each sample by its corresponding hyphal weight.

### Genomic DNA and RNA extraction

The *C. militaris* conidia were inoculated in PDB media for 3 days at 130 rpm and 25 °C. The hyphal pellets were washed with DEPC-treated water, and genomic DNA was extracted using the Monarch Genomic DNA Purification kit (New England Biolab, cat# T3010S). Total RNA was purified using the E.Z.N.A fungal RNA mini kit (Omega Bio-TeK, SKU R6840-01)—was used after TRIzol lysis (Thermo Fisher, cat # 15596026), and cDNA synthesis was performed using the ProtoScript® II First Strand cDNA Synthesis Kit (New England Biolab, cat# 6560S).

### Phylogenetic analysis

PCR amplification was performed using the primers ITS1 and ITS4 (Supplementary Table S15), and target DNA was amplified by OneTaq® 2X Master Mix with Standard Buffer (New England Biolab, cat# M0482S) under a thermal cycle of 94 °C for 5 min, followed by 35 cycles of 94 °C for 45 s, 52 °C for 1 min, 72 °C for 1.5 min, and a final extension at 72 °C for 5 min. The amplification products were purified with Qiaquick PCR Purification kits (Qiagen, cat # 28104) and then used directly for sequencing. Sequencing was performed using an ABI 3500 Series Genetic Analyzer (Thermo Fisher) and BigDye™ Terminator v3.1 Cycle Sequencing Kit (ThermoFisher, cat # 4337455). BioEdit software^41^ was utilized to examine the DNA sequence quality and accuracy as well as select unambiguous bases. Multiple sequences were aligned, and BLAST search and phylogenetic analysis were performed using MEGA version 11.0.13^42^. The FASTA sequences of the *C. militaris* DM1066^43^ and JLCY-LI819^44^ strains were also included in the phylogenetic analysis with the minimum-evolutionary method^45^ and interior-branch test^46^. The neighbor joining tree^47^ embedded on the NCBI website was used to explore the genetic relationships among *Ywt*, *Ydga* and other *C. militaris* strains.

### Transcriptome analysis

The transcriptome datasets from BioProject # PRJNA393201^48^ with BioSample # YCCZ1-YCCZ6^22,49^ were downloaded from the NCBI website using the SRA-tool kit^50^. The fastq data were checked using FastQC^51^, aligned to the *C. militaris* CM01 reference genome^52^ using HISAT2^53^, and counted using HTSeq-count^54^. Differential expression analysis was performed using EdgeR^55^, and gene ontology enrichment analysis was performed using the ClusterProfiler package in R^56^. All BioSamples, except for YCCZ3, had over 20 million reads with an overall alignment rate of at least 86%. YCCZ3 was excluded from further analysis. The expression patterns of the remaining samples were evaluated using hclust and plot functions in R. The plot showed that YCCZ5 (DG3) and YCCZ2 (DG1) clustered together and were farther from YCCZ1 (WT), while YCCZ4 (DG2) and YCCZ6 (DG4) were in the middle, between WT and the cluster of DG1 and DG3 (Supplementary Fig. S2). DG1 is the second generation of WT and can still develop fruiting bodies. Therefore, we considered DG3 as the “true” *C. militaris* degenerate strain because it was the fifth passage of the WT and had lost its ability to form fruiting bodies^22^. We compared it with the WT sample to identify differentially expressed genes.

### RT-qPCR

To validate the dysregulated genes identified by Next Generation Sequencing and associated with phenotypic degeneration, we performed RT-qPCR using primers designed with PrimerQuest from the idtdna.com website (Supplementary Table S15). Each RT-qPCR reaction had a total volume of 20 µL, containing cDNA, relevant primers, and the SYBR Green Real-time PCR Master Mix (Takara). Applied Biosystems™ 7500 Real-Time PCR Systems (Thermo Fisher) were used for RT-qPCR. Osh5 (CCM_00742—oxysterol binding protein, putative) was used as an internal control (reference gene) for normalization of the target gene expression and to correct for variation between samples. The thermal cycle for RT-PCR was as follows: 94 °C for 1 min, followed by 40 cycles of 94 °C for 45 s, 53 °C for 60 s, and 72 °C for 20 s. Melting curve analyses were performed at the end of each PCR reaction to ensure that only specific products were amplified. The comparative 2CT method^57^ was employed to calculate relative expression levels among the target genes.

### Statistical analysis

We reported the ranges, means, and standard deviations (SD) of the replicates for all the data. The graphs were generated using the ggplot2 package in R, and means and SD were manually added to improve visibility. To assess statistical significance, a one-tailed Student's t-test with an alpha level of 0.05 was conducted between the control and experimental groups using the ggsignif package in R. For the comparison of radical expansion, spore density, fruiting body weight, and glycerol concentration, a minimum of 5 biological replicates were utilized. For RT-qPCR analysis, three technical replicates were performed.

### Supplementary Information

Supplementary Information.

## Data Availability

All data generated or analyzed during this study are included in this published article (and its additional files). The RNAseq data associated with BioProject PRJNA393201 are deposited in NCBI databases by Yin at el^22^. Accession numbers of this dataset can be found on the BioProject page at the following link: https://www.ncbi.nlm.nih.gov/bioproject/?term=PRJNA393201.

## Abbreviations

MAPK: Mitogen activated protein kinases
*RT-qPCR*: Quantitative reverse transcription polymerase chain reaction
HOG: High-osmolarity glycerol
SF: Spore formation
PR: Pheromone response
FG: Filamentous growth
CWI: Cell wall integrity
ITS: Internal transcribed spacer
DG: Degenerate strain
WT: Wild-type
NAC: *N*-Acetyl cysteine
KCl: Potassium chloride
NaCl: Sodium chloride
PDA: Potatoes dextrose agarose
DC: Doi Chung meaning Control in English
