# Supplementary material for: Molecular mechanisms underlying phenotypic degeneration in Cordyceps militaris: insights from transcriptome reanalysis and osmotic stress studies
Source: Sci Rep. 2024 Jan 26;14:2231. doi: 10.1038/s41598-024-51946-3 (PMC10817986; doi:10.1038/s41598-024-51946-3)

SUPPLEMENTARY INFORMATION

Molecular Mechanisms Underlying Phenotypic Degeneration in *Cordyceps militaris*:

Insights from Transcriptome Reanalysis and Osmotic Stress Studies

Chinh Q. Hoang, Giang H Duong, Mai H. Tran, Tao V. Xuan, Tram B. Tran, Hang T. N. Pham

Table S1. ITS sequences used for phylogenetic analysis

>YWT.ITS1
TACCTATCGTTGCTTCGGCGGACTCGCCCAGCGCCTGGACGCGGGCCTGGGCGGCGGCCGTCGGGGGCCCCAAACACTGTA
TCTACCAGTTTTTCTGAATCCGCCGCAAGGCAAAACAAATGAATCAAAACTTTCAACAACGGATCTCTTGGCTCTGGCATCGATG
AAGAACGCAGCGAAATGCGATAAGTAATGTGAATTGCAGAATTCAGTGAATCATCGAATCTTTGAACGCACATTGCGCCCGCCA
GCATTCTGGCGGGCATGCCTGTTCGAGCGTCATTTCAACCCTCGACGTCCCCTGGGGGATGTCGGCGTTGGGG
>YDGA.ITS1
TACCTATCGTTGCTTCGGCGGACTCGCCCAGCGCCTGGACGCGGGCCTGGGCGGCGGCCGTCGGGGGCCCCAAACACTGTA
TCTACCAGTTTTTCTGAATCCGCCGCAAGGCAAAACAAATGAATCAAAACTTTCAACAACGGATCTCTTGGCTCTGGCATCGATG
AAGAACGCAGCGAAATGCGATAAGTAATGTGAATTGCAGAATTCAGTGAATCATCGAATCTTTGAACGCACATTGCGCCCGCCA
GCATTCTGGCGGGCATGCCTGTTCGAGCGTCATTTCAACCCTCGACGTCCCCTGGGGGATGTCGGCGTTGGGG
>NF.ITS1
GAGGGATCGTTACGAGTTTTCCACTCCCACCCTTTGTGAACATACCTATCGTTGCTTCGGCGGACTCGCCCAGCGCCTGGACG
CGGGCCTGGGCGGCGGCCGTCGGGGGCCCCAAACACTGTATCTACCAGTTTTTCTGAATCCGCCGCAAGGCAAAACAAATGA
ATCAAAACTTTCAACAACGGATCTCTTGGCTCTGGCATCGATGAAGAACGCAGCGAAATGCGATAAGTAATGTGAATTGCAGAA
TTCAGTGAATCATCGAATCTTTGAACGCACATTGCGCCCGCCAGCATTCTGGCGGGCATGCCTGTTCGAGCGTCA
>WT.ITS1
GACCAGCGGAGGGATCGTTACGAGTTTTCCACTCCCACCCTTTGTGACATACCTATCGTTGCTTCGGCGGACTCGCCCAGCGC
CTGGACGCGGGCCTGGGCGGCGGCCGTCGGGGGCCCCAAACACTGTATCTACCAGTTTTTCTGAATCCGCCGCAAGGCAAAA
CAAATGAATCAAAACTTTCAACAACGGATCTCTTGGCTCTGGCATCGATGAAGAACGCAGCGAAATGCGATAAGTAATGTGAATT
GCAGAATTCAGTGAATCATCGAATCTTTGAACGCACATTGCGCCCGCCAGCATTCTGGCGGGCATGCCTGTTCGAGCGTCA
Published sequences used as references
>MT525327.1 C. militaris JLCY-LI819
AACCTGCGGAGGGATCATTAACGAGTTTTCCAACTCCCAACCCTTTGTGAACATACCTATCGTTGCTTCGGCGGACTCGCCCAG
CGCCTGGACGCGGGCCTGGGCGGCGGCCGTCGGGGGCCCCAAACACTGTATCTACCAGTTTTTCTGAATCCGCCGCAAGGCA
AAACAAATGAATCAAAACTTTCAACAACGGATCTCTTGGCTCTGGCATCGATGAAGAACGCAGCGAAATGCGATAAGTAATGTG
AATTGCAGAATTCAGTGAATCATCGAATCTTTGAACGCACATTGCGCCCGCCAGCATTCTGGCGGGCATGCCTGTTCGAGCGTC
ATTTCAACCCTCGACGTCCCCTGGGGGATGTCGGCGTTGGGGACCGGCAGCACACCGCCGCCCCCGAAATGAAGTGGCGGC
CCGTCCGCGGCGACCTCTGCGTAGTACCCCAACTCGCACCGGGAACCCGACGTGGCCACGCCGTAAAACGCCCAACTCTGAA
CGTTGACCTCGGATCAGGTAGGAATACCCGCTGAACTTAAGCATATCA
>MT640261.1 C. militaris DM1066
CATTAATCAGGAACGAAAGTTAGGGGATCGAAGACGATCAGATACCGTCGTAGTCTTAACCATAAACTATGCCGACTAGGGATC
GGACGATGTTATTTTTTGACGCGTTCGGCACCTTACGAGAAATCAAAGTGCTTGGGCTCCAGGGGGAGTATGGTCGCAAGGCT
GAAACTTAAAGAAATTGACGGAAGGGCACCACCAGGGGTAAACCGCTTCGCGCAGCCGCAGTAGCTCTGCCCCAGAAAGCAG
CCCGAAAGGGTCAGTGGTGTTCCTGCCAGCGCTCCGGCGCGGCGAACTAATTGCTAGTCTCCTCTGGAGGCGACACCCTCAA
ATTGCGGGAAAATCCTAAAGCCAGTGTCACCAAGCCGGCGCCGAAAGGGCGTCGGTGGCCGGGGTAGCGACCTAGGGTACG
GTAAAAGCACACTGGATAATGCAATGGACGATCCGCAGCCAAGCCTCGTCGCCGCAGGGCACGGGGAAGGTTCAGAGACTAC
ATGGGGGTGGGTAGTGCACTGTCTGGGGGCTCCGGCCCCCGAGCGCCCGCTCACAACACTGCTTAAGATATAGTCCGGCCGT
GCTGGAAACAGCACGGGCTGGCAAACCCAGAAACGGGAGCCTGCGGCTTAATTTGACTCAACACGGGGAAACTCACCAGGTC
CAGACACAATGAGGATTGACAGATTGAGAGCTCTTTCTTGATTTTGTGGGTGGTGGTGCATGGCCGTTCTTAGTTGGTGGAGTG
ATTTGTCTGCTTAATTGCGATAACGAACGAGACCTTAACCTGCTAAATAGCCTGTATTGCTTTGGCAGTACGCTGGCTTCTTAGA
GGGACTATCGGCTCAAGCCGATGGAAGTTTGAGGCAATAACAGGTTGACCTACACAGGCCTGTAACAGTAGTCCTCGTTAAAAT
ATCTGCTAGTCCATGTAACGTCTCTGTTGGGGCAGTCCCCTTCTATCGGGCAGAGAGGATTCGAAAGAGTCCTAGTTACGGGC
GACACCACCTGGTACAGGGAACGCCGACCCAGGCACATCAGCAATGATGTACTGGAGGCCGATCCTGTGGCGAGCTCGGGTC
ACGCCGAGCCGTCGCAACGTGCGGAAAGGGGTGGGCCAGCATAACCTGCTGGCTTAAGGTACGTACTAATCCCATGGGAAAC
CATGCCCGTGCAGCAAGGCCCGATAGGCTGATCTGCACGGGGGCAGGCCACAGGCCTGCCGGCGTCGCCCGCGTATGCGGG
TGGACGAACAAATGCTGTGATGCCCTTAGATGTTCTGGGCCGCACGCGCGCTACACTGACGGAGCCAGCGAGTACTTCCTTGG
TCGAAAGGCTCGGGTAATCTTGTTAAACTCCGTCGTGCTGGGGATAGAGCATTGCAATTATTGCTCTTCAACGAGGAATCCCTA
GTAAGCGCAAGTCATCAGCTTGCGTTGATTACGTCCCTGCCCTTTGTACACACCGCCCGTCGCTACTACCGATCGAATGGCTCA
GTGAGGCGTCCGGACTGGCCCAGGGAGGTGGGCAACTACCCCCCCGGGCCGGGAAGCTCTCCAAACTCGGTCATTTAGAGG
AAGTAAAAGTCGTAACAAGGTCTCCGTTGGTGAACCAGCGGAGGGATCATTAACGAGTTTTCCAACTCCCAACCCTTTGTGAAC
ATACCTATCGTTGCTTCGGCGGACTCGCCCAGCGCCTGGACGCGGGCCTGGGCGGCGGCCGTCGGGGGCCCCAAACACTGT
ATCTACCAGTTTTTCTGAATCCGCCGCAAGGCAAAACAAATGAATCAAAACTTTCAACAACGGATCTCTTGGCTCTGGCATCGAT
GAAGAACGCAGCGAAATGCGATAAGTAATGTGAATTGCAGAATTCAGTGAATCATCGAATCTTTGAACGCACATTGCGCCCGCC
AGCATTCTGGCGGGCATGCCTGTTCGAGCGTCATTTCAACCCTCGACGTCCCCTGGGGGATGTCGGCGTTGGGGACCGGCAG
CACACCGCCGCCCCCGAAATGAAGTGGCGGCCCGTCCGCGGCGACCTCTGCGTAGTACCCCAACTCGCACCGGGAACCCGA
CGTGGCCACGCCGTAAAACGCCCAACTCTGAACGTTGACCTCGGATCAGGTAGGAATACCCGCTGAACTTAAGCATATCAA

Figure S1. The neighbor joining tree analysis of the genetic relationship among *Ywt, Ydga*, and reported *C. militaris* strains.

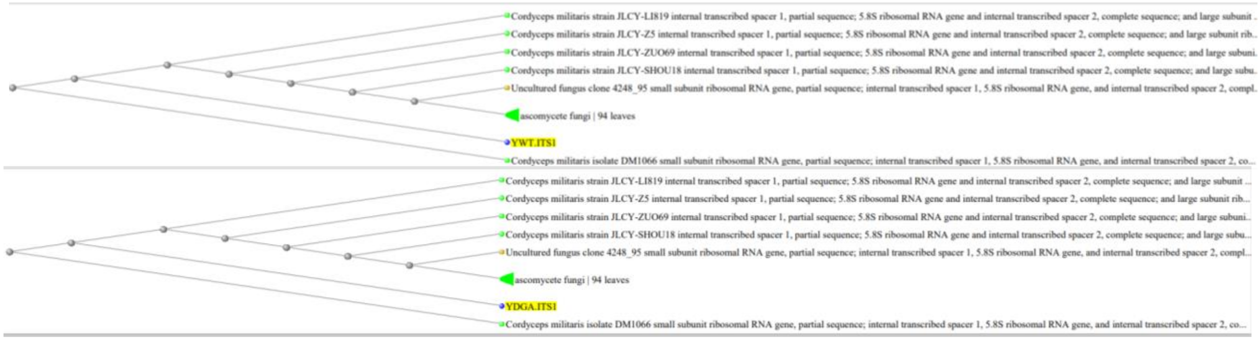

Table S2. Radical expansion of *C. militaris* strain *Ywt* vs. *Ydga* during development. The data are presented as ranges, means, and standard deviations, with a sample size of n = 5. SD refers to standard deviation. Sample ID indicates the biological replicates of strain names. Significance was determined using a one-tailed t-test for two independent means, with an alpha level of 0.05.

**6-day culture**				**12-day culture**				**16-day culture**			
**Sample ID**	**Diameter**	**Circle areas**	**T-test**	**Sample ID**	**Diameter**	**Circle areas**	**T-test**	**Sample ID**	**Diameter**	**Circle areas**	**T-test**
Ywt1	2,60	5,31		Ywt1	5,80	26,42		Ywt1	6,20	30,19	
Ywt2	2,40	4,52	*Mean: 5.08*	Ywt2	6,10	29,22	*Mean: 25.91*	Ywt2	6,40	32,17	*Mean: 31.18*
Ywt3	2,50	4,91	*SD: 0.46*	Ywt3	5,50	23,76	*SD:2.1*	Ywt3	6,20	30,19	*SD:0.99*
Ywt4	2,50	4,91		Ywt4	5,70	25,52		Ywt4	6,30	31,17	
Ywt5	2,70	5,73		Ywt5	5,60	24,63		Ywt5	6,40	32,17	
			**P = 0.0002**				**P = 0.0001**				**P = 0.00000 001**
Ydga1	2,00	3,14		Ydga1	4,60	16,62		Ydga1	4,80	18,10	
Ydga2	1,90	2,84		Ydga2	4,60	16,62		Ydga2	4,90	18,86	
Ydga3	2,00	3,14	*Mean: 3.02*	Ydga3	4,40	15,21	*Mean: 16.2*	Ydga3	4,80	18,10	*Mean: 17.66*
Ydga4	1,90	2,84	*SD:0.16*	Ydga4	4,70	17,35	*SD:0.95*	Ydga4	4,50	15,90	*SD:1.12*
Ydga5	2,00	3,14		Ydga5	4,40	15,21		Ydga5	4,70	17,35	

Table S3. Spore density of *C. militaris* strain *Ywt* vs. *Ydga* during development. The data are presented as ranges, means, and standard deviations, with a sample size of n = 5. SD refers to standard deviation. Sample ID indicates the biological replicates of strain names. Significance was determined using a one-tailed t-test for two independent means, with an alpha level of 0.05.

Sample ID	1st	2nd	3rd	4th	5th	Average	Dilution	Concentration	# ml	Total	Diameter	Area of a circle	density(10^6)/cm2	T-test
**6-day culture**														
Ywt1						19	1	4750000	2	9500000	2,6	5,30929	1,79	*Mean: 1.63*
Ywt2						12	1	3000000	2	6000000	2,4	4,52389	1,33	*SD:0.23*
Ywt3						14	1	3500000	2	7000000	2,5	4,90873	1,43	
Ywt4						18	1	4500000	2	9000000	2,5	4,90873	1,83	
Ywt5						20	1	5000000	2	10000000	2,7	5,72555	1,75	
														**P = 0.000007**
Ydga1	54	52	71	60	48	57	1	11850000	3.4	40290000	2	3,14159	12,82	
Ydga2	40	40	38	51	46	43	1	8950000	4	35800000	1,9	2,83528	12,63	
Ydga3	50	68	59	42	51	54	1	10350000	4	41400000	2	3,14159	13,18	*Mean: 13.12*
Ydga4	46	49	48	35	44	44,4	1	8620000	4	34480000	1,9	2,83528	12,16	*SD:1.02*
Ydga5	58	52	62	63	50	57	1	11650000	4	46600000	2	3,14159	14,83	
**12-day culture**														
Ywt1	46	45	53	45	43	46,4	1	11600000	8	92800000	6,2	30	3,07	*Mean: 2.68*
Ywt2	42	43	49	56	38	45,6	1	11400000	8	91200000	6,4	32	2,83	*SD:0.44*
Ywt3	35	26	35	26	33	31	1	7750000	8	62000000	6,2	30	2,05	
Ywt4	33	40	40	32	43	37,6	1	9400000	8	75200000	6,3	31	2,41	
Ywt5	37	39	42	38	40	39,2	1	9800000	9	88200000	6,1	29	3,02	**P = 0.00004**
Ydga1	136	224	160	152	156	165,6	1	41400000	7,6	314640000	4,7	17	18,14	
Ydga2	196	184	192	196	204	194,4	1	48600000	7,6	369360000	4,8	18	20,41	
Ydga3	184	176	160	260	252	206,4	1	51600000	7,6	392160000	4,6	17	23,60	*Mean: 19.85*
Ydga4	128	176	148	152	132	147,2	1	36800000	7,6	279680000	4,4	15	18,39	*SD:2.28*
Ydga5	130	160	168	150	140	149,6	1	37400000	7,6	284240000	4,4	15	18,69	
**16-day culture**														
Ywt1	46	45	53	45	43	46,4	1	11600000	8	92800000	6,2	30	3,07	*Mean: 2.48*
Ywt2	42	43	49	56	38	45,6	1	11400000	8	91200000	6,4	32,1699	2,83	*SD:0.46*
Ywt3	35	26	35	26	33	31	1	7750000	8	62000000	6,2	30,1907	2,05	
Ywt4	33	40	40	32	43	37,6	1	9400000	8	75200000	6,3	31,1724	2,41	
Ywt5	36	32	33	28	37	33,2	1	8300000	8	66400000	6,4	32,1699	2,06	**P = 0.0001**
Ydga1	136	224	160	152	156	165,6	1	31730000	7,6	241148000	4,7	17,3494	13,90	
Ydga2	196	189	192	196	204	195,4	1	39420000	7,6	299592000	4,8	18,0955	16,56	
Ydga3	184	176	160	260	252	206,4	1	43970000	7,6	334172000	4,6	16,6190	20,11	*Mean: 16.38*
Ydga4	128	176	148	152	132	147,2	1	29010000	7,6	220476000	4,4	15,2053	14,50	*SD:2.24*
Ydga5	139	166	145	155	235	168	1	35200000	7,6	267520000	4,5	15,9043	16,82	

Table S4. Biological pathways significantly enriched in downregulated genes along with their associated genes. *DE = Differentially Expressed.*

Pathway ID	Pathway names	GeneRatio	BgRatio	pvalue	p.adjust	qvalue	# of DE genes
cmt02010	ABC transporters - Cordyceps militaris	16/152	38/2057	1.97E-09	1.38E-07	1.35E-07	16
cmt04011	MAPK signaling pathway - yeast - Cordyceps militaris	16/152	63/2057	6.36E-06	0.000223	0.0002177	16
cmt00520	Amino sugar and nucleotide sugar metabolism - Cordyceps militaris	13/152	57/2057	0.000166	0.003872	0.00378511	13

No.	DE genes in ABC transporter	DE genes in MAPK pathway	DE genes in Amino sugar and nucleotide sugar metabolism
1	CCM_08739	CCM_05444	CCM_06535
2	CCM_03607	CCM_03130	CCM_08231
3	CCM_01696	CCM_06568	CCM_02966
4	CCM_02004	CCM_02415	CCM_00318
5	CCM_02386	CCM_07679	CCM_02965
6	CCM_06618	CCM_07878	CCM_07033
7	CCM_01467	CCM_08862	CCM_05430
8	CCM_00623	CCM_02506	CCM_08511
9	CCM_03253	CCM_09124	CCM_04624
10	CCM_04460	CCM_01444	CCM_01980
11	CCM_06149	CCM_07079	CCM_05688
12	CCM_03445	CCM_01361	CCM_04625
13	CCM_07363	CCM_08816	CCM_06973
14	CCM_08649	CCM_02193	
15	CCM_06538	CCM_05107	
16	CCM_08294	CCM_09157	

Table S5. The RT-PCR expression levels of genes associated with phenotypic degeneration. 2^∆Ct represents relative expression level in either *Ywt* or *Ydga* strains, which is normalized with the expression of *Osh5* genes. The suffixes 1, 2 and 3 represent the 1st, 2nd and 3rd biological replicates. The data are presented as ranges, means, and standard deviations, with a sample size of n = 3. SD refers to standard deviation. Significance was determined using a one-tailed t-test for two independent means, with an alpha level of 0.05.

Genes	Ywt-2^∆Ct1	Ywt-2^∆Ct2	Ywt-2^∆Ct3	Ydga-2^∆Ct1	Ydga-2^∆Ct2	Ydga-2^∆Ct3	T-test		
AbaA	0,28	0,27	0,29	0,58	0,60	0,61	mean: 0.28/0.60	SD: 0.01/0.02	**p = 0.00002**
Brla	0,33	0,34	0,35	0,75	0,74	0,75	mean: 0.34/0.75	SD: 0.01/0.01	**p = 0.0000009**
Osh5	1,00	1,00	1,00	1,00	1,00	1,00			
Gcy1	0,43	0,44	0,45	1,06	1,03	1,04	mean: 0,44/1,04	SD: 0.01/0.01	**p = 0.0000002**
GPP	0,48	0,50	0,49	1,00	0,97	0,98	mean: 0,49/0,98	SD: 0.01/0.01	**p = 0.00000004**
GDP	0,01	0,01	0,01	0,00	0,01	0,01	mean: 0,01/0,01	SD: 0/0	**p = 0.1**
Mcm1	0,12	0,13	0,14	0,05	0,03	0,04	mean: 0,13/0,04	SD: 0,01/0,01	**p = 0.0003**
Ste12	0,38	0,41	0,39	0,21	0,21	0,23	mean: 0,39/0,21	SD: 0,02/0,02	**p = 0.0003**
Cla4	0,46	0,57	0,52	0,69	0,70	0,71	mean: 0,52/0.7	SD: 0,06/0,02	**p = 0.02**
Ste20	0,32	0,41	0,38	0,90	0,88	0.9	mean: 0,37/0,89	SD: 0,05/0,01	**p = 0.001**

Table S6. The *C. militaris Ydga* strain has higher intracellular glycerol contents than that of *Ywt* strain. *OD = Optical Density at 413 nm.* The data are presented as ranges, means, and standard deviations, with a sample size of n = 5. SD refers to standard deviation. Sample ID indicates the biological replicates of strain names. Significance was determined using a one-tailed t-test for two independent means, with an alpha level of 0.05.

Sample ID	OD	Glycerol concentration	dilution	Total glycerol content	C. militaris wet weight	Glycerol content/g of C.militaris	T-test
ywt1	0,846	0,003453	1	0,003453	7,1	0,486338028	
ywt2	3,102	0,015861	1	0,015861	10,9	1,455137615	*Mean*: 1.09
ywt3	2,621	0,0132155	1	0,0132155	12	1,101291667	SD = 0.36
ywt4	3,484	0,017962	1	0,017962	15	1,197466667	
ywt5	4,017	0,0208935	1	0,0208935	17,2	1,214738372	**p = 0.004**
ydga1	4,064	0,021152	1	0,021152	10,2	2,07372549	
ydga2	4	0,0208	1	0,0208	10,4	2	
ydga3	4,337	0,0226535	1	0,0226535	11,6	1,952887931	*Mean*: 1.93
ydga4	4,023	0,0209265	1	0,0209265	11,9	1,758529412	SD = 0.12
ydga5	4,521	0,0236655	1	0,0236655	12,7	1,863425197	

Table S7. the CWI stressor represses the radical expansion and sporulation of *C. militaris* strain *Ywt vs. Ydga*. *PDA = Potatoes Dextrose Agar; CR = PDA + 200 ug/ml Congo Red***.** The data are presented as ranges, means, and standard deviations, with a sample size of n = 5. SD refers to standard deviation. Sample ID indicates the biological replicates of strain names. Significance was determined using a one-tailed t-test for two independent means, with an alpha level of 0.05.

Sample ID	Areas of Circles	T-test	Spore density (10^6)	T-test		Sample ID	Areas of Circles	T-test	Spore density (10^6)	T-test
Ywt.PDA1	22,90		1,21			Ydga.PDA1	15,21		44,33	
Ywt.PDA2	21,24	Mean: 23.26	1,01	Mean: 1.03		Ydga.PDA2	15,21	Mean: 15.35	44,72	Mean: 39.05
Ywt.PDA3	24,63	SD: 1.28	0,91	*SD: 0.11*		Ydga.PDA3	15,90	SD: 0.31	33,20	*SD: 5.31*
Ywt.PDA4	23,76		0,98			Ydga.PDA4	15,21		34,86	
Ywt.PDA5	23,76	p = 0.000003	1,05	p = 0.0003		Ydga.PDA5	15,21	P = 0.000001	38,14	**P = 0.01**
Ywt.CR1	13,20		0,61			Ydga.CR1	11,95		26,62	
Ywt.CR2	13,20		0,74			Ydga.CR2	11,34		27,51	
Ywt.CR3	15,21	Mean: 14.4	0,64	Mean: 0.65		Ydga.CR3	11,34	Mean: 11.23	31,21	*Mean*: 30.35
Ywt.CR4	15,21	SD: 1.1	0,70	*SD: 0.08*		Ydga.CR4	10,75	SD: 0.5	33,48	*SD: 3.13*
Ywt.CR5	15,21		0,53			Ydga.CR5	10,75		32,92	

Table S8. The effects of oxidative stress on the radical expansion and sporulation of *C. militaris* strain *Ywt* compared to *Ydga*. *PDA = Potatoes Dextrose Agar; H2O2 = PDA + 0.04% H2O2; NAC = PDA + 200 mM N-AcetylCysteine***.** The data are presented as ranges, means, and standard deviations, with a sample size of n = 5. SD refers to standard deviation. Sample ID indicates the biological replicates of strain names. Significance was determined using a one-tailed t-test for two independent means, with an alpha level of 0.05.

**Sample ID**	**Areas of Circles**	**T-test**	**Spore density (10^6)**	**T-test**		**Sample ID**	**Areas of Circles**	**T-test**	**Spore density (10^6)**	**T-test**
Ywt.PDA1	26,42		0,98			Ydga. PDA1	15,90		45,27	
Ywt.PDA2	25,52	Mean: 26.61	1,46	Mean: 1.3		Ydga. PDA2	16,62	Mean: 16.48	62,87	*Mean*: 60.49
Ywt.PDA3	28,27	*SD: 1.01*	1,41	*SD: 0.2*		Ydga. PDA3	17,35	*SD: 0.61*	69,08	*SD: 9.62*
Ywt.PDA4	26,42		1,43			Ydga. PDA4	15,90		57,64	
Ywt.PDA5	26,42		1,21			Ydga. PDA5	16,62		67,60	
**PDA vs. H2O2**		**p = 0.007**		**p < 0.001**				**p < 0.002**		**p = 0.1**
Ywt.H2O2.1	21,24		1,54			Ydga.H2O2.1	13,20		57,26	
Ywt.H2O2.2	24,63		1,94			Ydga.H2O2.2	13,20		71,14	
Ywt.H2O2.3	24,63	*Mean: 23.61*	1,94	Mean: 1.96		Ydga.H2O2.3	13,85	Mean: 13.46	86,40	*Mean*: 73.08
Ywt.H2O2.4	22,90	*SD: 1.52*	2,30	*SD: 0.28*		Ydga.H2O2.4	13,85	SD: 0.36	77,30	*SD: 10.06*
Ywt.H2O2.5	24,63		2,10			Ydga.H2O2.5	13,20		73,28	
**PDA vs. NAC**		**p = 0.00004**		**p = 0.08**				**p = 0.01**		**p = 0.2**
Ywt.NAC1	23,76		0,98			Ydga.NAC1	15,21		32,49	
Ywt.NAC2	23,76		1,06			Ydga.NAC2	15,21		44,98	
Ywt.NAC3	24,63	Mean: 23.93	1,27	*Mean*: 1.11		Ydga.NAC3	13,85	Mean: 15.08	45,12	*Mean*: 51.2
Ywt.NAC4	23,76	*SD: 0.39*	1,06	*SD: 0.12*		Ydga.NAC4	15,90	SD: 0.75	67,20	*SD: 15.06*
Ywt.NAC5	23,76		1,20			Ydga.NAC5	15,21		66,23	

Table S9. Hyperosmotic stressors suppress the radical expansion, but promotes sporulation of *C. militaris* strain *Ywt vs. Ydga*. *PDA = Potatoes Dextrose Agar; Nacl = PDA + 0.4 M Nacl; Kcl = PDA + 0.4 M Kcl.* The data are presented as ranges, means, and standard deviations, with a sample size of n = 5. SD refers to standard deviation. Sample ID indicates the biological replicates of strain names. Significance was determined using a one-tailed t-test for two independent means, with an alpha level of 0.05.

**Sample ID**	**Areas of Circles**	**T-test**	**Spore density (10^6)**	**T-test**		**Sample ID**	**Areas of Circles**	**T-test**	**Spore density (10^6)**	**T-test**
Ywt.PDA1	22,06		0,78			Ydga.PDA1	15,21		25,35	
Ywt.PDA2	24,63	*Mean*: 23.42	0,67	*Mean*: 0.68		Ydga.PDA2	16,62	*Mean*: 16.63	20,76	*Mean*: 24.79
Ywt.PDA3	22,90	*SD: 0.98*	0,53	*SD: 0.09*		Ydga.PDA3	15,90	*SD: 1.14*	29,14	*SD: 3*
Ywt.PDA4	23,76		0,69			Ydga.PDA4	18,10		24,18	
Ywt.PDA5	23,76		0,74			Ydga.PDA5	17,35		24,50	
**PDA vs. Nacl**		**P = 0.00000003**		**P = 0.000005**				**P = 0.0000007**		**P = 0.000001**
Ywt.Nacl1	9,62		4,86			Ydga.Nacl1	3,14		44,09	
Ywt.Nacl2	10,18		5,01			Ydga.Nacl2	3,14		49,18	
Ywt.Nacl3	9,62		5,79			Ydga.Nacl3	5,73		44,97	
Ywt.Nacl4	8,55	*Mean*: 9.41	5,03	*Mean*: 5.12		Ydga.Nacl4	4,91	*Mean*: 4.08	50,11	*Mean*: 47.26
Ywt.Nacl5	9,08	*SD: 0.62*	4,93	*SD: 0.38*		Ydga.Nacl5	3,46	*SD: 1.18*	47,93	*SD: 2.63*
**PDA vs. Kcl**		**P = 0.000000009**		**P = 0.000007**				**P = 0.000004**		**P = 0.008**
Ywt.Kcl1	8,04		5,63			Ydga.Kcl1	5,31		45,77	
Ywt.Kcl2	10,18		5,47			Ydga.Kcl2	5,31		41,81	
Ywt.Kcl3	9,62		6,32			Ydga.Kcl3	6,16		35,73	
Ywt.Kcl4	9,08	*Mean*: 9.09	6,54	*Mean*: 6.04		Ydga.Kcl4	5,73	*Mean*: 5.73	31,53	*Mean*: 37.22
Ywt.Kcl5	8,55	*SD: 0.84*	6,26	*SD:0.47*		Ydga.Kcl5	6,16	*SD: 0.42*	31,26	*SD: 6.41*

Table S10. Hyperosmotic stressors increase the intracellular glycerol contents of *C. militaris* strain *Ywt*. *PDA = Potatoes Dextrose Agar; Nacl = PDA + 0.4 M Nacl; Kcl = PDA + 0.4 M Kcl; Sor = PDA + 1 M Sorbitol. OD = Optical Density at 413 nm.* The data are presented as ranges, means, and standard deviations, with a sample size of n = 5. SD refers to standard deviation. Sample ID indicates the biological replicates of strain names. Significance was determined using a one-tailed t-test for two independent means, with an alpha level of 0.05.

Sample ID	OD	Glycerol content	Dilution	Total glycerol content	C. militaris wet weight	Glycerol content/g of C.militaris	T-test
Ywt.PDA1	2,456	0,0123	1	0,0123	8,5	1,448	*Mean*: 1.66
Ywt.PDA2	2,818	0,0143	1	0,0143	9,1	1,571	*SD:0.25*
Ywt.PDA3	3,358	0,0173	1	0,0173	9,1	1,898	
Ywt.PDA4	3,973	0,0207	1	0,0207	14,4	1,434	
Ywt.PDA5	2,777	0,0141	2	0,0281	14,4	1,954	
**PDA vs. Sor**							**p = 0.00002**
Ywt.Sor1	3,638	0,0188	1	0,0188	5,2	3,617	
Ywt.Sor2	3,046	0,0156	2	0,0311	6,7	4,643	*Mean*: 4.45
Ywt.Sor3	3,112	0,0159	2	0,0318	6,9	4,614	*SD: 0.47*
Ywt.Sor4	3,345	0,0172	2	0,0344	7,2	4,778	
Ywt.Sor5	3,212	0,0165	2	0,0329	7,2	4,574	
**PDA vs. Nacl**							**p = 0.0002**
Ywt.Nacl1	2,677	0,0135	3	0,0406	8,1	5,009	
Ywt.Nacl2	2,389	0,0119	4	0,0478	8,1	5,895	*Mean*: 5.47
Ywt.Nacl3	3,236	0,0166	3	0,0498	8,2	6,073	*SD: 0.8*
Ywt.Nacl4	3,284	0,0169	3	0,0506	8,3	6,094	
Ywt.Nacl5	2,947	0,0150	3	0,0450	10,5	4,289	
**PDA vs. Kcl**							**p = 0.00006**
Ywt.Kcl1	2,443	0,0122	3	0,0367	6,5	5,649	
Ywt.Kcl2	3,231	0,0166	3	0,0497	8,1	6,138	*Mean*: 5.29
Ywt.Kcl3	3,489	0,0180	3	0,0540	10,4	5,190	*SD: 0.65*
Ywt.Kcl4	3,307	0,0170	3	0,0510	11,5	4,432	
Ywt.Kcl5	3,107	0,0159	3	0,0477	9,5	5,018	

Table S11. Hyperosmotic stressors increase the intracellular glycerol contents of *C. militaris* strain *Ydga*. *PDA = Potatoes Dextrose Agar; Nacl = PDA + 0.4 M Nacl; Kcl = PDA + 0.4 M Kcl; Sor = PDA + 1 M Sorbitol. OD = Optical Density at 413 nm.* The data are presented as ranges, means, and standard deviations, with a sample size of n = 5. SD refers to standard deviation. Sample ID indicates the biological replicates of strain names. Significance was determined using a one-tailed t-test for two independent means, with an alpha level of 0.05.

Sample ID	OD	Glycerol content	Dilution	Total glycerol content	C. militaris wet weight	Glycerol content/g of C.militaris	T-test
Ydga.PDA1	3,1610	0,0162	2	0,0324	8,1	3,996	
Ydga.PDA2	2,8896	0,0147	2	0,0294	8,3	3,540	*Mean: 3.17*
Ydga.PDA3	2,8404	0,0144	2	0,0288	8,4	3,434	*SD: 0.7*
Ydga.PDA4	2,7218	0,0138	2	0,0275	11,4	2,416	
Ydga.PDA5	2,8208	0,0143	2	0,0286	11,7	2,447	
**PDA vs. Sor**							**p = 0.0003**
Ydga.Sor1	3,0619	0,0156	2	0,0313	4,0	7,820	
Ydga.Sor2	3,7059	0,0192	2	0,0384	4,4	8,719	
Ydga.Sor3	3,5516	0,0183	2	0,0367	5,5	6,667	*Mean*: 7.11
Ydga.Sor4	3,0516	0,0156	2	0,0312	5,0	6,233	*SD: 1.12*
Ydga.Sor5	3,5516	0,0183	2	0,0367	6,0	6,111	
**PDA vs. NaCl**							**p = 0.0008**
Ydga.Nacl1	2,7985	0,0142	4	0,0568	5,3	10,711	
Ydga.Nacl2	1,7036	0,0082	4	0,0327	5,4	6,052	*Mean*: 9.01
Ydga.Nacl3	2,5367	0,0128	4	0,0510	5,7	8,949	*SD: 1.77*
Ydga.Nacl4	2,4367	0,0122	4	0,0488	5,0	9,762	
Ydga.Nacl5	2,8367	0,0144	4	0,0576	6,0	9,601	
**PDA vs. Kcl**							**p = 0.0001**
Ydga.Kcl1	3,9778	0,0207	4	0,0827	6,5	12,725	
Ydga.Kcl2	4,2072	0,0219	4	0,0878	6,2	14,155	*Mean*: 12.23
Ydga.Kcl3	4,0186	0,0209	4	0,0836	6,1	13,706	*SD: 1.86*
Ydga.Kcl4	2,4433	0,0122	4	0,0490	4,6	10,642	
Ydga.Kcl5	3,2014	0,0164	4	0,0656	6,6	9,944	

Table S12. Hyperosmotic stress suppresses the radical expansion, but increases sporulation of *C. militaris* strain *Wt, Nt, Ywt* and *Ydga*. *PDA = Potatoes Dextrose Agar; Kcl = PDA + 0.4M Kcl.* The data are presented as ranges, means, and standard deviations, with a sample size of n = 5. SD refers to standard deviation. Sample ID indicates the biological replicates of strain names. Significance was determined using a one-tailed t-test for two independent means, with an alpha level of 0.05.

**Sample ID**	**Areas of Circles**	**T-test**	**Spore density (10^6)**	**T-test**		**Sample ID**	**Areas of Circles**	**T-test**	**Spore density (10^6)**	**T-test**
Wt.PDA1	28,27		4,80			Nf.PDA1	27,34		1,53	
Wt.PDA2	25,52	*Mean: 26.8*	4,09	*Mean: 5.56*		Nf.PDA2	28,27	*Mean: 26.4*	2,38	*Mean: 1.83*
Wt.PDA3	26,42	*SD: 1.3*	6,40	*SD: 1.07*		Nf.PDA3	25,52	*SD: 1.14*	1,79	*SD: 0.34*
Wt.PDA4	28,27		6,52			Nf.PDA4	26,42		1,56	
Wt.PDA5	27,34	**p = 0.000001**	6,00	**p = 0.00000002**		Nf.PDA5	26,42	**P = 0.00004**	1,87	**P = 0.0001**
Wt.Kcl1	15,21		31,75			Nf.Kcl1	12,57		23,46	
Wt.Kcl2	15,21		31,94			Nf.Kcl2	13,85		26,37	
Wt.Kcl3	14,52	*Mean: 14*	28,77	*Mean: 30.06*		Nf.Kcl3	13,20	*Mean: 12.4*	24,29	*Mean: 22.9*
Wt.Kcl4	13,85	*SD: 1*	28,68	*SD: 1.64*		Nf.Kcl4	12,57	*SD: 0.55*	17,87	*SD: 3.15*
Wt.Kcl5	13,85		29,14			Nf.Kcl5	12,57		22,53	
Ywt.PDA1	29,22		3,18			Ydga.PDA1	17,35		18,14	
Ywt.PDA2	28,27	*Mean: 27*	3,23	*Mean: 2.9*		Ydga.PDA2	18,10	*Mean: 16.2*	20,41	*Mean: 19.94*
Ywt.PDA3	27,34	*SD: 1.58*	2,27	*SD: 0.39*		Ydga.PDA3	16,62	*SD: 1.3*	23,60	*SD: 2.28*
Ywt.PDA4	26,42		2,85			Ydga.PDA4	15,21		18,39	
Ywt.PDA5	25,52	**p = 0.000000005**	2,98	**p = 0.0001**		Ydga.PDA5	15,90	**P = 0.00001**	18,68	**P = 0.003**
Ywt.Kcl1	12,57		28,97			Ydga.Kcl1	10,18		34,35	
Ywt.Kcl2	11,95		24,67			Ydga.Kcl2	11,34		50,22	
Ywt.Kcl3	13,20	*Mean: 12.2*	19,94	*Mean: 24*		Ydga.Kcl3	10,18	*Mean: 10*	31,06	*Mean: 38.7*
Ywt.Kcl4	13,20	*SD: 0.84*	23,39	*SD: 3.28*		Ydga.Kcl4	9,62	*SD: 0.71*	41,55	*SD: 7.48*
Ywt.Kcl5	12,57		23,02			Ydga.Kcl5	10,75		36,30	

Table S13. Hyperosmotic stress increases the intracellular glycerol contents of *C. militaris* strain *Wt, Nt, Ywt* and *Ydga*. *PDA = Potatoes Dextrose Agar; Kcl = PDA + 0.4M Kcl*. *OD = Optical Density at 413 nm.* The data are presented as ranges, means, and standard deviations, with a sample size of n = 5. SD refers to standard deviation. Sample ID indicates the biological replicates of strain names. Significance was determined using a one-tailed t-test for two independent means, with an alpha level of 0.05.

Sample ID	OD	Glycerol contents	Glycerol content/g of C.militaris	T-test	Sample ID	OD	Glycerol contents	Glycerol content/g of C.militaris	T-test
Wt.PDA1	0,745	0,003	0,442		Nf.PDA1	2,887	0,015	0,944	
Wt.PDA2	0,864	0,004	0,511	*Mean:0.46*	Nf.PDA2	2,863	0,015	0,798	*Mean:0.82*
Wt.PDA3	0,607	0,002	0,408	*SD:0.05*	Nf.PDA3	3,865	0,020	0,806	*SD:0.07*
Wt.PDA4	0,707	0,003	0,430		Nf.PDA4	3,020	0,015	0,778	
Wt.PDA5	0,907	0,004	0,523	**p = 0.000002**	Nf.PDA5	3,210	0,016	0,800	**P < 0.00003**
Wt.Kcl1	4,013	0,021	7,196		Nf.Kcl1	3,120	0,016	3,137	
Wt.Kcl2	4,147	0,022	8,351		Nf.Kcl2	3,270	0,017	3,144	
Wt.Kcl3	3,829	0,020	6,111	*Mean:7.44*	Nf.Kcl3	3,288	0,017	2,483	*Mean:2.94*
Wt.Kcl4	3,615	0,019	7,664	*SD:0.05*	Nf.Kcl4	3,380	0,017	3,037	*SD:0.27*
Wt.Kcl5	3,815	0,020	7,913		Nf.Kcl5	3,488	0,018	2,907	
Ywt.PDA1	0,330	0,001	0,070		Ydga.PDA1	1,300	0,006	0,124	
Ywt.PDA2	0,359	0,001	0,080	*Mean:0.08*	Ydga.PDA2	1,560	0,007	0,559	*Mean:0.61*
Ywt.PDA3	0,387	0,001	0,089	*SD:0.008*	Ydga.PDA3	2,309	0,011	0,763	*SD:0.29*
Ywt.PDA4	0,367	0,001	0,088		Ydga.PDA4	2,159	0,011	0,846	
Ywt.PDA5	0,357	0,001	0,087	**p = 0.00005**	Ydga.PDA5	2,209	0,011	0,788	**p < 0.000002**
Ywt.Kcl1	3,114	0,016	3,309		Ydga.Kcl1	2,791	0,014	3,462	
Ywt.Kcl2	3,186	0,016	3,051		Ydga.Kcl2	2,611	0,013	3,016	
Ywt.Kcl3	4,987	0,026	3,041	*Mean:3.20*	Ydga.Kcl3	2,364	0,012	2,559	*Mean:2.99*
Ywt.Kcl4	4,387	0,023	3,474	*SD:0.18*	Ydga.Kcl4	2,464	0,012	2,959	*SD:0.32*
Ywt.Kcl5	4,687	0,025	3,146		Ydga.Kcl5	2,624	0,013	2,957	

Table S14. Hyperosmotic stress represses the fruiting body development of *C. militaris* strain *Wt, Nt, Ywt*. *DC = FBM (Fruiting Body Media); Kcl = FBM + 0.4 M Kcl***.** The data are presented as ranges, means, and standard deviations, with a sample size of n >/= 7. SD refers to standard deviation. Sample ID indicates the biological replicates of strain names and the corresponding cultured media. Significance was determined using a one-tailed t-test for two independent means, with an alpha level of 0.05.

**Sample ID**	**FB dry weight**	**T-test**	**Sample ID**	**FB dry weight**	**T-test**	**Sample ID**	**FB dry weight**	**T-test**
Ywt.DC1	3,08		Wt.DC1	2,44		Nf.DC1	2,38	
Ywt.DC2	3,15		Wt.DC2	2,67		Nf.DC2	2,53	*Mean*: 3.2
Ywt.DC3	3,47	*Mean*: 3.58	Wt.DC3	2,53	*Mean*: 2.73	Nf.DC3	3,23	*SD: 0.57*
Ywt.DC4	3,62	*SD: 0.35*	Wt.DC4	3,03	*SD: 0.51*	Nf.DC4	3,31	
Ywt.DC5	3,69		Wt.DC5	2,21		Nf.DC5	3,36	
Ywt.DC6	3,71		Wt.DC6	2,48		Nf.DC6	4,02	
Ywt.DC7	3,73		Wt.DC7	3,75	**p = 0.004**	Nf.DC7	3,58	**p = 0.002**
Ywt.DC8	4,16	**p = 0.000001**	Wt.Kcl1	1,58		Nf.Kcl1	1,93	
Ywt.Kcl1	1,5		Wt.Kcl2	2,16		Nf.Kcl2	2,06	
Ywt.Kcl2	1,55		Wt.Kcl3	0,71		Nf.Kcl3	2,27	
Ywt.Kcl3	1,68		Wt.Kcl4	0,48	*Mean*: 1.52	Nf.Kcl4	2,57	*Mean*: 2.16
Ywt.Kcl4	1,68		Wt.Kcl5	1,22	*SD: 0.75*	Nf.Kcl5	2,68	*SD: 0.36*
Ywt.Kcl5	2,24	*Mean*: 1.98	Wt.Kcl6	2,46		Nf.Kcl6	1,85	
Ywt.Kcl6	2,2	*SD: 0.43*	Wt.Kcl7	2		Nf.Kcl7	1,76	
Ywt.Kcl7	2,3							
Ywt.Kcl8	2,69							

Table S15. RT-PCR and ITS primer sequences.

No.	Primer ID	Primer Sequences
1	CCM_05107_STE20F	AGTGTCACCCTCGTCGATAA
2	CCM_05107_STE20R	GTAGGCGGCTTAGAAGGAATG
3	CCM_02629-CLA4F	CTCAAGGCCCTGTACCTTATTG
4	CCM_02629-CLA4R	CACAGACAGACAGACAGGAAAG
5	CCM_03292_Mcm1F	CAAGCGCAAGGCTGGTATTAT
6	CCM_03292_Mcm1R	TTGGAGTTTGGGTGTGGTAAAG
7	CCM_01444 _STE12F	CTCTGCCGAACCTGCTTATATC
8	CCM_01444 _STE12R	TGGACCATGCCGTTCATAC
9	CCM_03540 _GPD1F	GTGCAGATGTGGGTGTTTGA
10	CCM_03540 _GPD1R	CTGGTTGATGATGCTGGTGAG
11	CCM_06603-GPPF	GGTCGACGCAGCATTGATA
12	CCM_06603-GPPR	TCGCGAGATGGGCATAGA
13	CCM_01682-Gcy1F	GACCAAAGGGAAACTTGGAAAC
14	CCM_01682-Gcy1R	CTGCCAGATGCTCCAGATAC
15	CCM_05444_AbaAF	TGCCTATGACATCCACGATAC
16	CCM_05444_AbaAR	GTCTTTGCCGAGCACATTAG
17	CCM_08959 _BrlAF	CCTCAAGAAAGATGGCGGAAA
18	CCM_08959 _BrlAR	GTGTCTCTTTAGGTGCTCGTTG
19	CCM_00742-Osh5F	CCCAGCTAAGAAGAACTCCAAA
20	CCM_00742-Osh5R	TCGAGCGGATGTTGCTTATC
21	ITS1	CTTGGTCATT TAGAGGAAGTAA
22	ITS4	TCCTCCGCTTATTGATATG

Figure S2. Cluster dendrogram of RNAseq data.

(BioSample # YCCZ1 (WT), YCCZ2 (DG1), YCCZ4 (DG2), YCCZ5 (DG3), YCCZ6 (DG4))

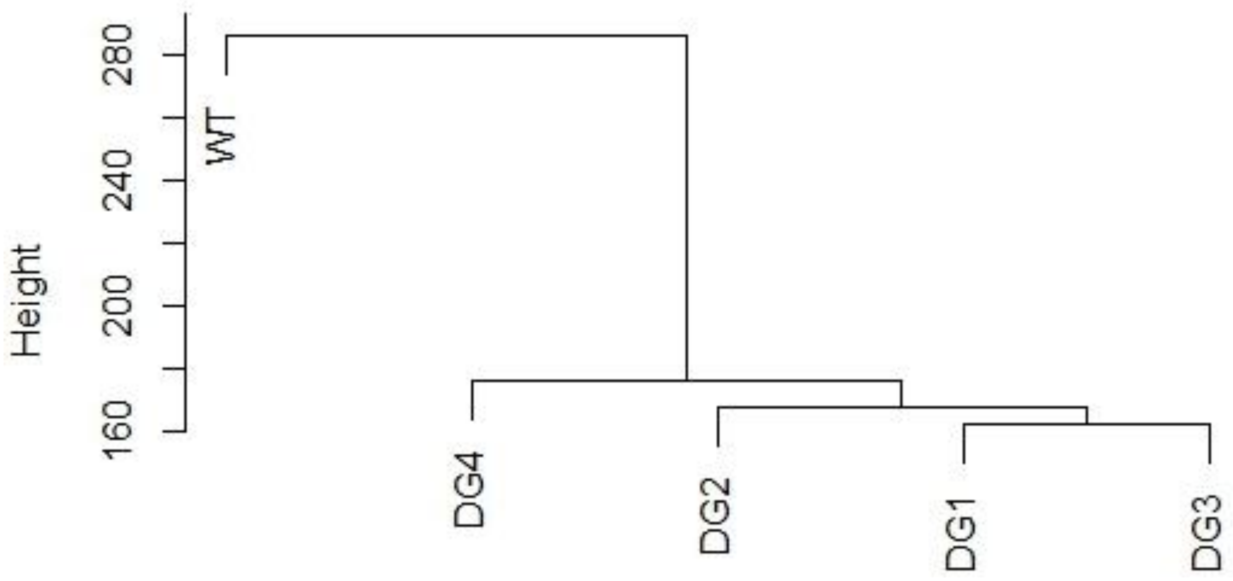

Supplement: Supplementary file 1 — Supplementary Information. [file 41598_2024_51946_MOESM1_ESM.pdf]
